# 1252. Penicillin Allergy Reassessment for Treatment Improvement (PARTI): A Dental Office Tool to Support Appropriate Penicillin Allergy Labeling

**DOI:** 10.1093/ofid/ofad500.1092

**Published:** 2023-11-27

**Authors:** Ashlan J Kunz Coyne, Dana Holger, Erinne Kennedy, Mackenzie Connell, Juliann Binienda, Christopher Giuliano, Elaine Bailey

**Affiliations:** University of Kentucky, Detroit, Michigan; Nova Southeastern University, Fort Lauderdale, Florida; Kansas City University, Joplin, Kansas; University of Florida, Gainesville, Florida; Wayne State University, Detroit, Michigan; Wayne State University Eugene Applebaum College of Pharmacy and Health Sciences, Detroit, Michigan; Michigan Antibiotic Resistance Reduction (MARR) Coalition, Detroit, Michigan

## Abstract

**Background:**

Dentists routinely review patient allergy histories and prescribe penicillin. Dental visits offer an opportunity to identify patient candidates for PCN allergy reassessment. Aim: to collect and evaluate clinician and patient feedback on a PCN Allergy Reassessment for Treatment Improvement (PARTI) tool created for patient-clinician communication about PCN allergy labels, and ultimately, to delabel patient EHR records, as appropriate.

**Figure:**
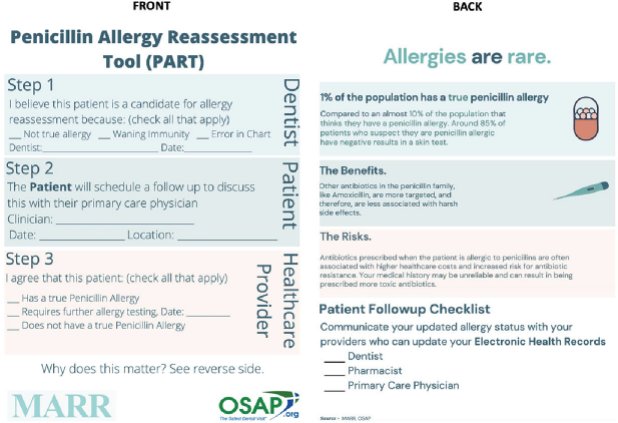

**Methods:**

A mixed methods pilot study was conducted January-March 2023. We administered a semi-quantitative questionnaire to interdisciplinary clinicians and performed semi-structured focus groups of patients with a PCN allergy label for tool feedback. The questionnaire and focus group guide were developed to focus on the three tool components: (1) steps for PCN allergy reassessment, (2) general patient-centered PCN allergy information, and (3) a follow-up checklist for updating EHR allergy records. Deductive thematic analysis was used for the focus group data.

**Results:**

In total, 50 clinicians completed the questionnaire and 15 patients participated in focus groups. Both groups included individuals from 5/5 U.S. regions. Survey respondents included mostly pharmacists (30%) and dentists (20%). PARTI steps 1, 2, and 3 were rated as “very important” or “important” for 81%, 67% and 93% of clinicians, respectively. Inclusion of the patient information section was supported by 100% of clinicians and 94% thought the PARTI tool was at an appropriate literacy level. Stated barriers to using the PARTI tool included patient follow-through and provider comfort with evaluating PCN allergies. Regarding the PARTI tool itself, focus group participants indicated that the tool was a conversation starter, easy to understand, included helpful patient-centered allergy information, and that the follow-up checklist for EHR record updates was useful. Stated patient barriers to the PARTI tool included patient follow-through and clinician hesitancy to update allergy records.

**Conclusion:**

Patients and clinicians were receptive to using the PARTI tool for PCN allergy reassessment. Data herein will be used to address clinician and patient feedback to further develop the PARTI tool in preparation for its use in a national, multidisciplinary pilot study.

**Disclosures:**

**All Authors**: No reported disclosures

